# Points from Hospital Reports

**Published:** 1924-11

**Authors:** 


### POINTS FROM HOSPITAL REPORTS.

### Royal Northern Hospital.

This group of hospitals (Holloway, City Road, and Southgate) treated in 1923 4,285 in-patients and 36,175 out-patients, both figures being higher than in 1922. A neurological department, a light department, and orthopædic sessions were inaugurated during the year. Unfortunately, these institutions are hampered with a debt of over £50,000, which necessitated the closure of 60 beds. Consequently, a long waiting list of patients cannot be dealt with, and this heavy debt also prevents developments required to meet the needs of the growing population of North London. Gifts would therefore be particularly welcome at the present time.

### Glasgow Royal Maternity and Women's Hospital.

The normal accommodation of this Hospital is 108 beds, and when we state that the average number of in-patients resident daily throughout 1923 was 123, the pressure under which the Hospital worked will be appreciated. Turning to finance we find that the income was less than in 1922 by £319, and that subscriptions decreased by £224. The deficit on the year was £307. In view of the commercial and industrial conditions prevailing during 1923 these adverse figures are matters for congratulation rather than alarm. As the Report says, nobody would have been surprised had the results been far worse. We notice with special commendation the great success of the Ladies' Auxiliary. Its 40 Town Divisions and 68 County Divisions contributed respectively £673 and £989. Were we asked to say what in our opinion is the dominating characteristic of this Report, the answer would be, enthusiasm. In one place the Directors speak of their Hospital as "the finest Hospital of its kind in the world," and a few lines further on they say, "It is most encouraging to see that of all the subscriptions those from the workers have increased, an experience which is unique and cannot be claimed by any other Hospital." Without a doubt this is the way to inspire and sustain the spirit of perseverance in a Hospital's supporters.

### Royal Westminster Ophthalmic Hospital.

The year 1923, in the words of the Report, "marks an important epoch in the history of the Hospital, in the granting by His Majesty the King, of a Royal Charter of Incorporation to the Institution. The Hospital under this Charter has now a legal entity, thus relieving Trustees and others of personal liability." The Charter is also, we venture to add, a badge of dignity not without practical advantage. Owing to the closing of the Wards for painting and cleaning for a longer time than usual, the number of in-patients was smaller than in 1922, while the number of out-patients was considerably higher. The income was well maintained, and more than sufficient to meet the somewhat increased expenditure. Patients' payments furnished practically a quarter of the income. The Hospital has 42 beds.

### City of London Maternity Hospital.

The work of this, the oldest of the lying-in hospitals as they were formerly called, was carried on in full activity in 1923. The number of in-patients was slightly greater, and that of out-patients considerably smaller than in 1922. As might have been expected from this the average cost of an in-patient was less and that of an out-patient more than in the preceding year. An increase of £539 in expenditure was more than counterbalanced by one of £1,188 in income. The year closed with a balance to the good of £1,175 on the hospital account, after including as hospital expenditure a deficit of £1,714 on the Midwifery Training School account. The interest on a loan of £14,830 from the bankers is a heavy charge, and efforts are to be made to reduce this debt.

### Paddington Green Children's Hospital.

An increase in work over the previous year, a slight but not proportionate increase of expenditure, and a small increase of income, are the main features of the operations of 1923. It is pleasing to be able to point to a material reduction in the average cost per in-patient per week, and to a decrease also, small though it is, in the average cost of an out-patient. An endeavour, attended with not very material success, was made in the last quarter of the year to obtain contributions towards paying off the large debt (£13,500) to the bankers. The amount received was £223, and when we state that the expenditure of the year exceeded the income by £1,340, it will be seen that the net result of the year's financial transactions is a further retrogression of £1117. The revision and alphabetical arrangement of the table of contents would repay the little trouble it would occasion.

### General Infirmary, Salisbury.

Apart from an increase in the work of this infirmary of 141 beds, an improvement in the income resulting in a small excess of income over expenditure, and a reduction in the latter, the outstanding feature of the year was the visit of the late Sir Napier Burnett, who, having inspected the institution at the invitation of the President and Committee, furnished a report on its work and administration, containing much sound and helpful advice, which the Committee express the intention of acting on as soon as funds will permit. The report is a most interesting and methodical compilation, providing a ready answer to every reasonable inquiry. It is furnished with an excellent table of contents.

### Queen Charlotte's Maternity Hospital.

This hospital has changed its title from "Lying-in" to "Maternity" during the year. It has also decided to admit women, for the first time, to its Committee of Management; surely an appropriate and wise, if somewhat belated, change. "Queen Charlotte's" had a good financial year in 1923, as despite a charge of £326 for interest on loan, and a still heavier one (£1,589) in respect of the year's deficiency on the Midwifery Training School account, there was an excess of total income over total expenditure of £2,464. It seems to us that the cost of the education and training of midwives, so highly important to the nation, should not be a charge on the charitable funds of the Maternity Hospitals, already straitened as they are. The hospital contains 75 beds, and the number of patients attended, both in the wards and at their own homes, was greater than in 1922. There were material reductions in expenditure during the year, and hence in the average cost of the patients in both departments.

### Royal Alexandra Infirmary, Paisley.

Three years ago this hospital was faced with a deficit of £7,334, which has now happily been reduced to £850. We trust that the current year will see it wiped off entirely. The work was well maintained during 1923; the Garden Fête was again a great success, the receipts amounting to the substantial sum of £3,203. We do not find stated the number of beds available; the average occupied was 181·7. The report has no index.

### Manchester Babies' Hospital.

The work of this admirable little hospital was actively and very successfully carried on during 1923. Its success is shown very strikingly by the unprecedentedly low rate of mortality among the little patients—7·7 compared with a previous lowest of 16—which is attributable to the institution of "Nursing Mothers." The statistics of patients relate to the calendar year 1923, while the accounts are for the year ending March 31, 1924. A deficiency of £209 on a total of £6,525 is no very serious matter. Comparative statistics, either of patients or finance, are not much in evidence, and it is therefore not easy to note the changes from year to year. We have not succeeded in ascertaining the number of cots available; nor do we see an index to the Report. A scheme of extension is about to be launched, plans for the alteration of the present hospital and the building of new wards having been submitted to and been approved by the Ministry of Health.

### Dewsbury General Infirmary.

This modern hospital (founded 1876) had a busy and prosperous year in 1923. The number of in-patients was 1,022, compared with 998 in 1922; the income was augmented by £644, while the increase in expenditure was only £407. The average daily number of in-patients under treatment was 42. We do not find the number of beds available stated An index would improve the report.

